# Predictors of Conduction Disturbances Requiring New Permanent Pacemaker Implantation following Transcatheter Aortic Valve Implantation Using the Evolut Series

**DOI:** 10.3390/jcm12144835

**Published:** 2023-07-22

**Authors:** Mahmoud Abdelshafy, Ahmed Elkoumy, Hesham Elzomor, Mohammad Abdelghani, Ruth Campbell, Ciara Kennedy, William Kenny Gibson, Simone Fezzi, Philip Nolan, Max Wagener, Shahram Arsang-Jang, Sameh K. Mohamed, Mansour Mostafa, Islam Shawky, Briain MacNeill, Angela McInerney, Darren Mylotte, Osama Soliman

**Affiliations:** 1 Discipline of Cardiology, Galway University Hospital, SAOLTA Healthcare Group, Health Service Executive, H91 YR71 Galway, Ireland; mahmoud.abdelshafy@universityofgalway.ie (M.A.); aelkoumy@universityofgalway.ie (A.E.); hesham.elzomor@universityofgalway.ie (H.E.); missruthc@gmail.com (R.C.); c.kennedy40@nuigalway.ie (C.K.); billykgibson@gmail.com (W.K.G.); fezzisimone@gmail.com (S.F.); philipnolan92@gmail.com (P.N.); max.wagener@gmail.com (M.W.); briain.macneill@icloud.com (B.M.); angela_mcinerney@hotmail.com (A.M.); 2 CORRIB Core Lab, University of Galway, H91 V4AY Galway, Ireland; shahram.arsang-jang@universityofgalway.ie (S.A.-J.); sameh.kamaleldin@gmail.com (S.K.M.); 3 Department of Cardiology, Al-Azhar University, Cairo 11311, Egypt; m.abdelghani.nl@gmail.com (M.A.); mans_aref@yahoo.com (M.M.); islam012@hotmail.com (I.S.); 4 Islamic Center of Cardiology and Cardiac Surgery, Al-Azhar University, Cairo 11651, Egypt; 5 Department of Cardiology, Amsterdam UMC, Amsterdam Cardiovascular Sciences, University of Amsterdam, 1081 HV Amsterdam, The Netherlands; 6 Discipline of Medicine, Clinical Science Institute, University of Galway, H91 YR71 Galway, Ireland; 7 CÚRAM Centre for Medical Devices, H91 TK33 Galway, Ireland

**Keywords:** TAVI, conduction disturbance, computed tomography, pacemaker implantation, membranous septum

## Abstract

(1) Background: Conduction disturbance requiring a new permanent pacemaker (PPM) after transcatheter aortic valve implantation (TAVI) has traditionally been a common complication. New implantation techniques with self-expanding platforms have reportedly reduced the incidence of PPM. We sought to investigate the predictors of PPM at 30 days after TAVI using Evolut R/PRO/PRO+; (2) Methods: Consecutive patients who underwent TAVI with the Evolut platform between October 2019 and August 2022 at University Hospital Galway, Ireland, were included. Patients who had a prior PPM (*n* = 10), valve-in-valve procedures (*n* = 8) or received >1 valve during the index procedure (*n* = 3) were excluded. Baseline clinical, electrocardiographic (ECG), echocardiographic and multislice computed tomography (MSCT) parameters were analyzed. Pre-TAVI MSCT analysis included membranous septum (MS) length, a semi-quantitative calcification analysis of the aortic valve leaflets, left ventricular outflow tract, and mitral annulus. Furthermore, the implantation depth (ID) was measured from the final aortography. Multivariate binary logistic analysis and receiver operating characteristic (ROC) curve analysis were used to identify independent predictors and the optimal MS and ID cutoff values to predict new PPM requirements, respectively; (3) Results: A total of 129 TAVI patients were included (age = 81.3 ± 5.3 years; 36% female; median EuroSCORE II 3.2 [2.0, 5.4]). Fifteen patients (11.6%) required PPM after 30 days. The patients requiring new PPM at 30 days were more likely to have a lower European System for Cardiac Operative Risk Evaluation II, increased prevalence of right bundle branch block (RBBB) at baseline ECG, have a higher mitral annular calcification severity and have a shorter MS on preprocedural MSCT analysis, and have a ID, as shown on the final aortogram. From the multivariate analysis, pre-TAVI RBBB, MS length, and ID were shown to be predictors of new PPM. An MS length of <2.85 mm (AUC = 0.85, 95%CI: (0.77, 0.93)) and ID of >3.99 mm (area under the curve (AUC) = 0.79, (95% confidence interval (CI): (0.68, 0.90)) were found to be the optimal cut-offs for predicting new PPM requirements; (4) Conclusions: Membranous septum length and implantation depth were found to be independent predictors of new PPM post-TAVI with the Evolut platform. Patient-specific implantation depth could be used to mitigate the requirement for new PPM.

## 1. Introduction

Transcatheter aortic valve implantation (TAVI) is an established treatment option for older patients with symptomatic severe aortic stenosis, irrespective of operative risk [1,2]. New conduction disturbances, particularly new left bundle branch block (LBBB) and permanent pacemaker (PPM) implantation, have been associated with increased all-cause mortality and heart failure hospitalization at one year [3]. The incidence of conduction disturbance and the need for new PPM remains frequent, despite the advancements in device technology and implantation techniques [4]. The direct compression of conduction tissue by the transcatheter heart valve (THV), resulting in local ischemia, oedema, and haemorrhage, may explain the injury of the often calcific conductive system [5].

The rate of new PPM was five times more frequent with the self-expanding first-generation CoreValve system (Medtronic, Dublin, Ireland) (25–28%) compared with balloon-expanding valves (5–7%) (SAPIEN and SAPIEN XT; Edwards Lifesciences, Irvine, CA, USA) [6,7]. Recent studies have shown that the PPM rates obtained using the SAPIEN 3 and SAPIEN 3 Ultra valves can be as low as 4.4–6.5% [2,8]. The introduction of newer-generation CoreValve systems (Evolut R/Pro/Pro+) with novel features, such as the ability to recapture and reposition, has been associated with a lower rate of new PPM [1]. When combined with increasing operator experience and novel imaging and implantation techniques, the rate of new PPM with contemporary self-expanding platforms is lower, but the data are less robust, falling short of a continuous technology/technique dynamicity.

Previously identified predictors of new PPM post-TAVI are older age, right bundle branch block (RBBB) on baseline electrocardiogram (ECG), higher mean aortic valve gradient, calcification including left ventricular outflow tract (LVOT) or mitral annulus, membranous septum (MS) length and implantation depth (ID) [4,9]. Multislice computed tomography (MSCT) can identify the MS, which serves as an anatomical landmark and represents the distance between the aortic annulus and the atrioventricular conduction system. The ID plays a vital role as a modifiable predictor of new PPM and is the focus of ongoing investigations on TAVI using the Evolut platform. It appears that implanting the device higher in relation to the length of the MS can reduce the likelihood of post-TAVI PPM risk [10,11,12,13].

In this study, we aimed to investigate the rate and predictors of conduction disturbance requiring a new PPM after TAVI with the Evolut R/PRO/PRO+ systems in contemporary clinical practice.

## 2. Materials and Methods

### 2.1. Study Design and Patient Population

This is a single-centre, retrospective, observational study. Consecutive patients who underwent TAVI with the Medtronic Evolut R/PRO/PRO+ system between October 2019 and August 2022 at University Hospital Galway, Ireland, were reviewed for inclusion eligibility. Patients with prior PPM undergoing a valve-in-valve procedure, or patients who received >1 valve during the index procedure, were excluded. The study complied with the ethical guidelines of the Declaration of Helsinki and was approved by the institutional ethical committee.

### 2.2. TAVI Procedure

Pre-procedure workup included baseline ECG, transthoracic echocardiography (TTE), and multislice computed tomography (MSCT). The heart team determined eligibility for TAVI in all cases. Standard in-hospital care post-TAVI included daily ECG until hospital discharge. Echocardiography was performed in all cases post-TAVI. TAVI procedure was performed in accordance with the instructions for use and the hospital’s standard procedure. Valve release was performed under fast or rapid pacing, with an optimal final ID of 3–5 mm. Local anaesthesia was used, except in exceptional circumstances when general anaesthesia was used. Pre- and/or post dilatation was performed at the discretion of the operating team.

### 2.3. MSCT Analysis

The pre-TAVI MSCT was analysed according to the recommendations of the Society of Cardiovascular Computed Tomography [14]. The reconstruction and analysis were performed using 3mensio Structural Heart software program version 10.3 (Pie Medical Imaging, Maastricht, The Netherlands). Calcification of the valvular apparatus at aortic cusps and left ventricular outflow tract was visually graded as none = 0, mild = 1, moderate = 2, and severe = 3. The index of annular eccentricity was calculated as [1 − (minimum diameter/maximum diameter)] and the degree of oversizing by area as [(prosthesis area/annulus area − 1) × 100%] and by perimeter as [(prosthesis perimeter/annulus perimeter − 1) × 100%]. Mitral annular calcification (MAC) was defined as the presence of dense calcium deposits at the base of mitral leaflets, grade 0 = no MAC, grade 1 = mild MAC affecting ≤ 25% of the annulus, grade 2 = moderate MAC affecting 25–50% of the annulus, grade 3 = severe MAC affecting ≥50% of the annulus [15]. The MS length measurement was performed by an independent imaging cardiologist blinded to post-TAVI outcomes. For a standardized analysis, the cursor in the perpendicular co-planar view was placed at the intersection of the non-coronary and right coronary cusp. MS was defined on this perpendicular co-planar view as the thinnest part of the interventricular septum between LVOT and the right atrium from the nadir of the non-coronary cusp to the tip of the muscular interventricular septum [12,13].

### 2.4. ID Measurement

The ID was determined on the final aortogram post-TAVI and was measured as the depth from the edge of the THV frame up to the nadir of the non-coronary cusp (NCC) [12].

### 2.5. ECG Data

A 12-lead ECG was collected at three timepoints: baseline (within 24 h before the procedure), immediately after the procedure (post-TAVI), and at hospital discharge. The diagnosis of conduction abnormalities was classified according to the recommendations of the American Heart Association/American College of Cardiology Foundation/Heart Rhythm Society (AHA/ACCF/HRS) for the standardization and interpretation of ECGs [16]. PR interval and QRS duration were analysed for each ECG to calculate the change (delta) from baseline to post-TAVR and the change from baseline to discharge.

### 2.6. Clinical Data and TAVI Clinical Outcome

The clinical data were obtained from a prospectively managed, dedicated database within Galway University Hospital. Clinical outcomes were defined based on the Valve Academic Research Consortium-3 (VARC-3) consensus document [17].

### 2.7. Study Outcome

The primary outcome of our study was to investigate the predictors of new PPM post-Evolut implantation at 30 days, while the secondary outcome was to explore the changes in the PR interval and QRS duration from baseline to post-TAVI and pre-discharge.

### 2.8. Statistical Analysis

Categorical variables were presented as numbers and percentages. Continuous variables were reported as mean ± standard deviation (SD) or median and interquartile range (IQR) as appropriate. The Shapiro–Wilk test was used to test the normality of continuous variables. Baseline patient characteristics, comorbidities, ECG data, echocardiographic data, MSCT data, procedural and post-procedural parameters were compared between those requiring and not requiring a new PPM. Continuous data were compared using Student’s *t*-test (normality) or Mann–Whitney U test (non-normality). Categorical data were compared using chi-square test or Fisher’s exact test. Independent predictors of new PPM were determined using binary logistic regression and the backward method for variable selection. Odds ratios (ORs), along with their corresponding 95% confidence intervals (CIs), were used to report the results. The variables included in the univariable analysis were the European System for Cardiac Operative Risk Evaluation II (EuroSCORE II), right bundle branch block (RBBB), membranous septum (MS) length, more than/equal moderate mitral annular calcification (MAC), implantation depth (ID) and the difference between the MS length and the ID. Parameters with a *p*-value ≤ 0.01 in univariate analyses were included in multivariate analyses. The variables included in the multivariable analysis were RBBB, MS length and ID. A *p*-value of less than 0.05 in multivariate analysis was considered statistically significant. Receiver operating characteristic (ROC) curve analysis was employed to identify the preprocedural and procedural parameters that best predict new PPM and to determine the optimal cut-off value for that/those parameter(s). All statistical analyses were performed using SPSS Statistics for Windows, version 25.0 (SPSS, Inc., Chicago, IL, USA).

## 3. Results

**Figure 1:**
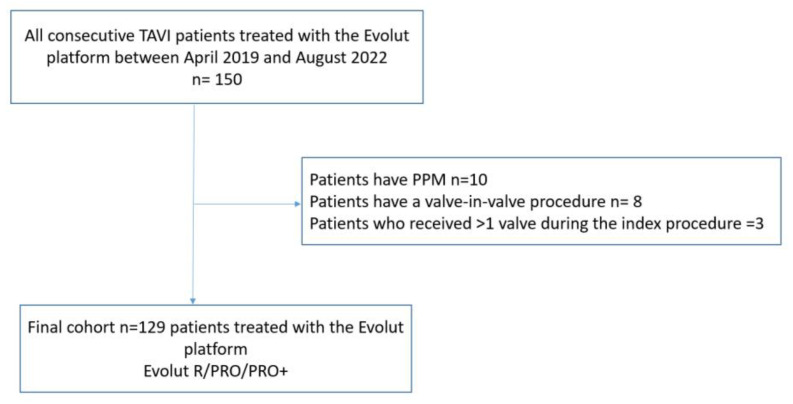
Study Flow chart.

Between April 2019 and August 2022, 150 patients were treated with the Medtronic Evolut platform. After the exclusion of patients who had PPM at baseline (*n* = 10), patients who had a valve-in-valve procedure (*n* = 8), and patients who received >1 valve during the index procedure (*n* = 3), the final cohort included 129 patients (Figure 1).

### 3.1. Baseline Characteristics

The average age was 81.3 (±5.3) years, and one-third (36%) were female. The median European System for Cardiac Operative Risk Evaluation (EuroSCORE) II was 3.2 [2.0, 5.4]. All underwent transfemoral TAVI with local anaesthesia, except for two patients with general anaesthesia. Balloon pre-dilation was used in 69%, and balloon post-dilation was performed in 40%. The measurement of implantation depth was only feasible in 106 patients. The baseline demographic, clinical, ECG Echocardiographic and MSCT characteristics are detailed in Table 1, and procedural variables post-TAVI complications are displayed in Table 2.

### 3.2. Conduction Disturbance

**Figure 2:**
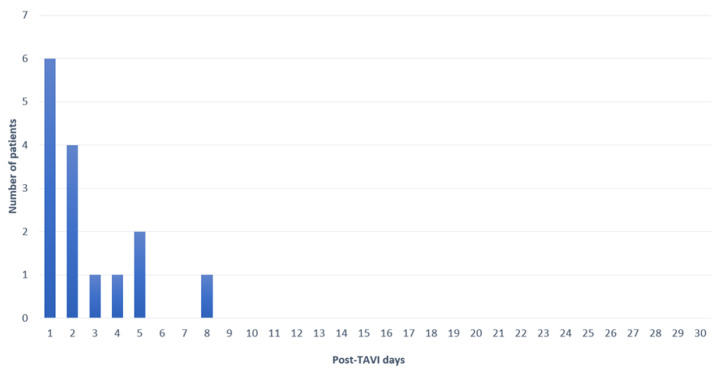
Time to new PPM implantation.

The rate of new PPM was 10% (13/129) at discharge and 11.6% (15/129) at 30 days, which was unchanged at one year. Seven of these fifteen patients (47%) had a pre-existing RBBB. All PPMs were inserted due to complete heart block, except for one patient with new LBBB (QRS duration = 179 millisecond (msec)) and first-degree AV block (PR duration = 330 msec). The median time until new PPM was 2 days [1, 3.5], as detailed in Figure 2.

### 3.3. Predictors of New PPM

The baseline clinical characteristics of patients with and without new PPM were similar, except for EuroSCORE II, which was lower in patients with new PPM (1.9 [1.7, 3.2] vs. 3.3 [2.1, 5.4], *p* = 0.008). Patients with new PPM were also more likely to have RBBB (47% vs. 8%, *p* < 0.001), shorter MS length (1.5 [1.1, 2.5] vs. 3.1 [2.3, 4], *p* = 0.002), a higher rate of ≥moderate MAC (60% vs. 33%, *p* = 0.047) and a deeper ID (4.4 [4.1, 5.7] vs. 3.6 [2.6, 4.1], *p* < 0.001). Moreover, the difference between the MS length and the ID was significantly greater in patients who required PPM (−3.9 ± 1.5 vs. −0.3 ± 2.4, *p* < 0.001).

### 3.4. Multivariate Predictors of New PPM

In the multivariate model, pre-existing RBBB, MS length, and implant depth, were independent predictors of new PPM Table 3.

**Figure 3:**
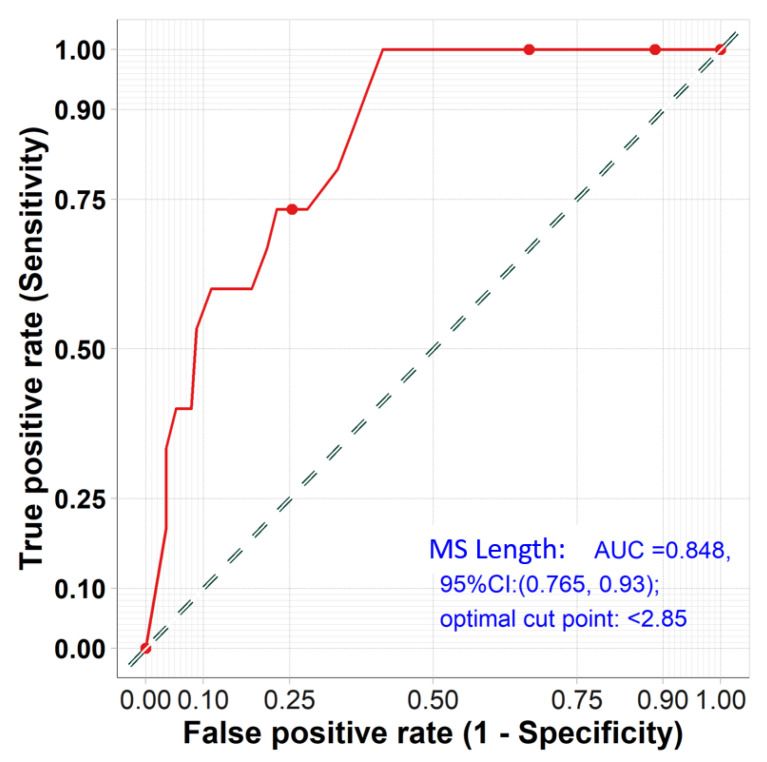
ROC results of the predictability power of membranous septum length for prediction of PPM.

**Figure 4:**
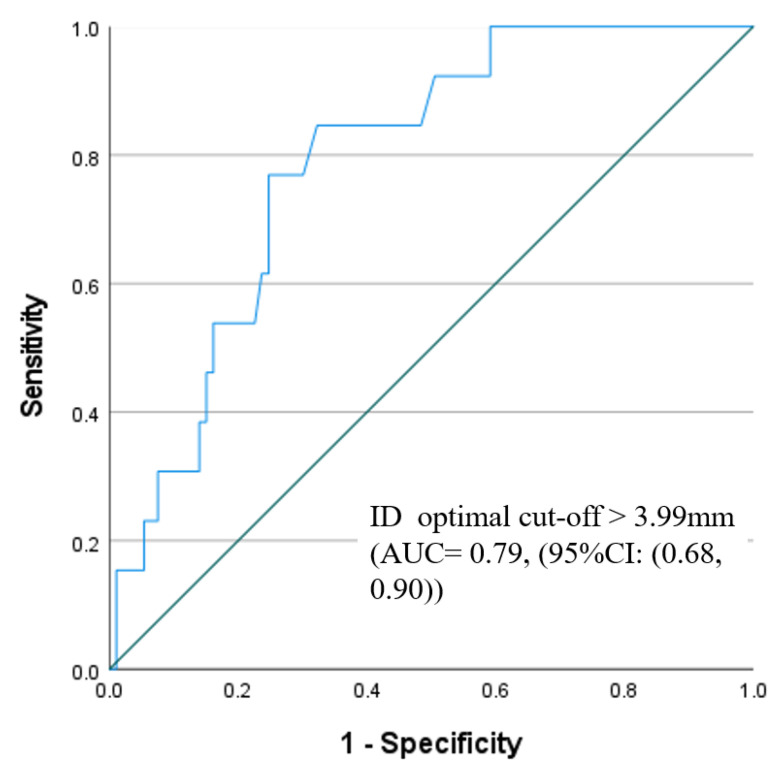
ROC results of the predictability power of implantation depth for prediction of PPM.

An MS length of <2.85 mm (AUC = 0.85, (95%CI: (0.77, 0.93) and ID of >3.99 mm (AUC = 0.79, (95%CI: (0.68, 0.90)) were found to be the optimal cut-offs by ROC analysis for predicting new PPM requirements at 30 days, as shown in Figure 3 and Figure 4.

### 3.5. PR Interval and QRS Duration Changes from Baseline to Post-Procedure and Discharge

**Figure 5:**
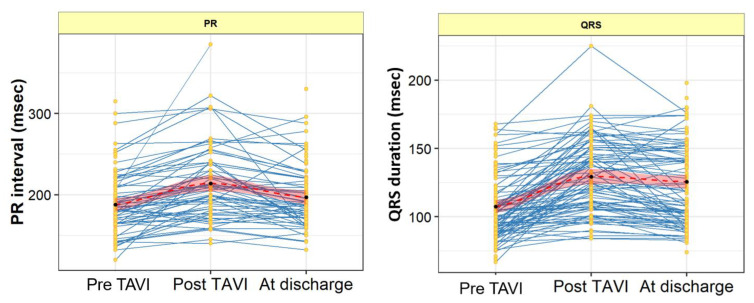
PR interval and QRS duration measurements pre-TAVI, post-TAVI and pre-discharge. The blue line represents the changes observed in each patient, while the red line depicts the average changes observed in all patients. Abbreviations: msec = millisecond; TAVI = transcatheter aortic valve implantation.

PR and QRS duration were calculated at three timepoints: immediately pre-TAVI, immediately post-TAVI and at discharge. The 15 patients who required new PPM post-TAVI were excluded from this analysis. The PR interval was prolonged post-TAVI [208 (178, 240) vs. 182 (164, 209) msec, *p* = 0.002], which was recovered at discharge [188 (171, 219) msec vs. 182 (164, 209) msec, *p* = 0.064]. On the other hand, the QRS duration was prolonged post-TAVI [127 (107, 150) vs. 101 (89, 119) msec, *p* < 0.001], which continued at discharge [125 (100, 150) msec, *p* < 0.001], as shown in Table 4 and Figure 5.

### 3.6. Procedural and Short-Term Outcomes

Procedural and in-hospital death occurred in 0% and 2% of patients, respectively. In-hospital deaths were attributed to stroke, right ventricular failure, and intestinal ischemia.

## 4. Discussion

The present study explored the predictors of new PPM in contemporary TAVI patients receiving Evolut platforms (R/PRO/PRO+). The main findings are as follows:

(1) At 30 days, the rate of new PPM implantation was 11.6%.
(2) On multivariate analysis, pre-existing RBBB, MS length, and ID were found to be the strongest predictors of new PPM.
(3) The optimal membranous septum length cut-off to predict new PPM was <2.85 mm AUC = 0.85, (95% CI: 0.77–0.93) while the optimal implantation depth cut-off was >3.99 mm and AUC = 0.79 (95% CI: 0.68–0.90).
(4) Detailed ECG analysis showed significant prolongation of the PR interval and QRS duration post-TAVI. The PR interval prolongation recovered pre-discharge, while QRS duration persisted until discharge compared to the baseline measurements.

Conduction abnormalities remain a significant hurdle to successful TAVI implantation. The close relation between His bundle and the left bundle branch to the aortic annulus explains this phenomenon. The conduction system injury is likely due to inflammation, oedema, or ischemia, which occur during TAVI implantation [5]. The His bundle course may be one of three anatomical variations: 50% penetrate the right side of the ventricular septum, 30% penetrate the left side and, infrequently, it courses under the membranous septum just below the endocardium (20%) [18]. These anatomical variations may explain the complexity of conduction disturbance predictions.

The rate of new PPM in our study was 11.6%, which is consistent with new PPM rates in the studies on newer-generation Evolut platforms that have been published to date [11,13], but less than the rate of new PPM in the Evolut Low Risk Trial [1]. These data are interesting, as our cohort would be considered low-risk, with a median EuroSCORE II of 3.2. This difference may be explained by the use of older-generation devices in the Evolut Low Risk trial (CoreValve and Evolut R), while Evolut R accounted for ~30% of the valves included in this study, with the majority of implants being Evolut Pro/Pro+. Our study, therefore, adds weight to the observation of a steady decline in new PPM requirements with successive iterations of the Medtronic Evolut family of devices. The adoption of COT and high ID in our cohort may be contributed to the lower PPM rate.

Pre-existing RBBB has been recognized as the most consistent predictor of new PPM implantation and, again, our study affirms this finding. The other predictors, e.g., MAC severity, MS length and implantation depth, and its relation to the MS length, were frequently identified in other studies [4,9]. The INTERSECT registry analyzed the effect of MS length on pacemaker requirements post-TAVI among 1811 patients, utilizing various TAVI devices. The study revealed that MS length was a significant predictor of PPM for all TAVI platforms, except for the ACURATE neo [19].

All these predictors are non-modifiable, except the implantation depth. Jilaihawi and colleagues [13] proposed that the high PPM achieved with the Evolut platform can be alleviated when aiming for a pre-release ID that is less than the MS length. The new PPM rate at 30 days was significantly lower in their prospective cohort using the suggested approach (3% vs. 9.7%, *p* = 0.035). Indeed, in our study, an ID greater than the MS length was also found to occur more frequently in those requiring a new PPM. The same approach, using a high deployment technique, was applied by Sammour et al. [20] for the implantation of balloon-expandable SAPIEN 3 valve, resulting in a significant reduction in the 30-days PPM post-TAVI (5.5% vs. 13.1%, *p* < 0.001). In a recently published meta-analysis on the use of MS length as a predictor of PPM after TAVI [21] and its interaction with the ID, including 18 studies, it was found that a short MS length and low difference between the MS length and the ID were associated with a higher risk of PPM post-TAVI.

Changes in the implantation technique are already underway with the Evolut family of devices. The wide variability in new the PPM requirements across previous studies with these platforms suggests the need for a standardization of implantation techniques. The incidence of new PPM post-TAVI in the Evolut Low Risk Trial, for example, ranged from 1.6% to 26.2% at the four highest implanting sites in the study [22]. The ongoing post-market Optimize PRO study (NCT04091048) aims to standardize implantation techniques using the cusp overlap view, paying particular attention to the implantation depth (targeting 3–5 mm). An interim analysis of North American sites found that the rate of new PPM implantations at 30 days was 9.8%, which significantly decreased to 5.8% when using the cusp overlap technique(COT) [23].

Use of the COT was numerically higher in patients who did not require a new PPM but statistically non-significant (67% vs. 47%, *p* = 0.16), which could be due to the relatively small sample size in our cohort.

On the other hand, the continuous improvement in the devices and their delivery systems led to a significant decrease in major periprocedural complications, including new PPM [7,10]. Initially, the original Medtronic CoreValve platform was approved for clinical use in Europe in 2007, followed by Evolut R in 2014, Evolut PRO and Evolut PRO+, and finally Evolut FX.

Evolut FX received FDA approval in August 2021 but is not yet approved in Europe. The Evolut FX has a more flexible delivery system to assist in the steering of the valve through complex anatomies and is equipped with three radiopaque markers to enhance visualization and improve position accuracy and commissural alignment [24]. Furthermore, the delivery system has an optimized stability layer for more predictable deployment. However, the initial results of first in human (FIH) [25] showed no statically significant difference between the Evolut FX (*n* = 43) and Evolut PRO+ (*n* = 378) regarding the rate of new PPM or new LBBB (7% vs. 11.2%, *p* = 0.78 and 16.3% vs. 10.6%, *p* = 0.20, respectively). Of note, the Evolut FX cohort had a significantly higher implantation (ID at NCC was 2.5 ± 2.3 vs. 3.4 ± 2.3, *p* = 0.016 and at LCC was 2.5 ± 2.3 vs. 3.4 ± 2.3, *p* = 0.016, respectively) with a higher rate of commissural alignment (93% vs. 80.2%; *p* = 0.039, respectively). Evidently, this study was a retrospective reporting the initial experience in a few numbers of patients treated with Evolut FX, which needs to be confirmed in prospective multicentre randomized studies.

There have been limited studies assessing the impact of TAVI on the cardiac electrical properties of patients who do not require a PPM after the procedure. In our study, both PR interval and QRS duration were significantly prolonged post-procedure in comparison to the baseline. The PR prolongation recovered while the QRS widening persisted at discharge. This is in contrast to other studies, which showed that the PR prolongation persisted at discharge [26,27]. After a six-month follow-up of 182 patients who underwent TAVI, it was observed that, while the QRS widening continued, the PR prolongation did not persist [26].

Predicting the need for new PPM following TAVI is possible with the presence of pre-existing RBBB, a short membranous septum and the presence of MAC on MSCT, which was significant in our study. Systematic measurement of the MS length during pre-procedure planning, aiming for a patient-specific implant depth, may be an important evolution in the implantation technique for these devices. These data should guide the procedural planning, and the discussion of the risk of new PPM should be integrated into the informed consent process with patients and during the institutional heart team discussion. Furthermore, it should be integrated into procedural planning, including device selection, implantation height, pre- and post-balloon dilation, choice of pacing strategy during the procedure, and the duration of post-procedure telemetry monitoring [9,28]. Finally, as a high THV implantation can potentially impede future access to the coronary arteries or render TAV-in-TAV procedures more challenging in a proportion of patients [11], the balance between avoiding a new PPM and facilitating future procedures should be carefully weighed on a case-by-case basis.

There are several important limitations. Our study is a single-centre retrospective study with a relatively small sample size, bearing the limitations inherent to this design. The average age of our patients was 81.3 years, and only 36% were female. It is important to consider this context when interpreting the study results. The measurement of ID from the final aortography may be affected by the angle of acquisition or the amount of injected contrast. Similarly, the presence of localized calcification or a narrow sinus makes the identification of the annular plane difficult. Post-TAVI MSCT can be the best option to obtain a precise ID measurement, which was not carried out in our study. Finally, we did not investigate the PPM dependence or the recovery of conduction after PPM implantation in our study.

## 5. Conclusions

In this single-centre retrospective study, the rate of new PPM implantation post-TAVI with the Evolut platform was 11.6% at 30 days. Membranous septum length and implantation depth were independent predictors of new PPM. A customized implantation depth based on the membranous septum length could mitigate the new PPM rate.

## Figures and Tables

**Table 1:** Baseline demographic, clinical, ECG, echocardiographic and MSCT characteristics.

	All Patients *n* = 129	PPM *n* = 15	No PPM *n* = 114	*p* Value
**Baseline characteristics**				
Age (years)	81.3 ± 5.3	81.7 ± 4.3	82.1 ± 5.3	0.36
Female, *n* (%)	46 (36%)	2 (13%)	44 (38.6%)	0.08
Body Mass Index (kg/m^2^)	26.8 [24.1, 31.1]	30 [23.7, 33.8]	26.8 [24.1, 30.7]	0.35
Hypertension, *n* (%)	99 (77%)	13 (87%)	86 (75%)	0.51
Diabetes, *n* (%)	43 (33%)	7 (47%)	36 (32%)	0.25
Dyslipidemia, *n* (%)	73 (57%)	12 (80%)	61 (54%)	0.058
NYHA class ≥ III, *n* (%)	76 (59%)	6 (40%)	70 (61.4%)	0.16
COPD, *n* (%)	22 (17%)	2 (13%)	20 (18%)	>0.999
Previous MI, *n* (%)	19 (15%)	2 (13%)	17 (15%)	>0.999
Prior CVA	14 (11%)	4 (27%)	10 (9%)	0.059
Glomerular filtration rate (mL/min/1.73 m^2^)	61.1 ± 16.7	59.1 ± 15.4	55.6 ± 20.3	0.42
EuroSCORE II	3.2 [2.0, 5.4]	1.9 [1.7, 3.2]	3.3 [2.1, 5.4]	0.008
**Baseline ECG**				
Atrial Fibrillation, *n* (%)	28 (22%)	2 (13%)	26 (23%)	0.52
RBBB	16 (12%)	7 (47%)	9 (8%)	<0.001
LBBB	12 (10%)	0 (0%)	12 (11%)	0.35
1st-degree AV block	32 (25%)	4 (27%)	28 (25%)	0.92
PR interval	183 [164, 209.5]	178 [156, 207.5]	184 [164, 212]	0.57
QRS duration	101 [89, 119]	120 [89, 140]	101 [89, 113]	0.18
**Echocardiographic data**				
LVEF ≤ 40%	33 (26%)	3 (20%)	30 (26%)	0.75
Mean AoV gradient (mmHg)	54.1 ± 32.4	60.1 ± 15.7	53.3 ± 34.1	0.45
Peak AoV gradient (mmHg)	80.8 ± 20.5	89.1 ± 18.3	79.6 ± 20.7	0.45
**MSCT characteristics**				
Bicuspid morphology	26 (20%)	5 (33%)	21 (18%)	0.18
Annulus diameter (mm)	25.3± 2.5	26.3± 2.7	25.2 ± 2.4	0.08
Annular eccentricity index	0.25 ± 0.06	0.27 ± 0.05	0.24 ± 0.06	0.17
Perimeter-derived annulus diameter (mm)	25.5 ± 2.2	26.5 ± 2.6	25.3 ± 2.3	0.09
Area-derived annulus diameter (mm)	24.9 ± 2.2	25.9 ± 2.7	24.8 ± 2.3	0.07
Annulus perimeter (mm)	80.4 ± 7.1	82.9 ± 8.1	79.9 ± 7.5	0.14
Annulus area (mm^2^)	493.1 ± 88.1	530.5 ± 104.9	487.9 ± 90.9	0.09
LCA height (mm)	16.3 ± 3.3	16.1 ± 4.2	16 ± 3.2	0.93
RCA height (mm)	18.9 ± 3.5	18.8 ± 3.8	18.7 ± 3.6	0.88
Aortic root angulation ≥ 49	56 (43%)	8 (53%)	48 (42%)	0.42
Membranous septum length (mm)	3 [2.1, 3.8]	1.5 [1.1, 2.5]	3.1 [2.3, 4]	<0.001
AoV calcification ≥ moderate	106 (82%)	14 (93%)	92 (81%)	0.30
LVOT calcification ≥ moderate	31 (24%)	4 (27%)	27 (24%)	0.75
MAC ≥ moderate	46 (36%)	9 (60%)	37 (33%)	0.047

Data presented as frequency and (percentage), mean ± standard deviation or median [interquartile range]. Abbreviations: AoV = aortic valve; AV block = atrioventricular block; COPD = chronic obstructive airway disease; CVA = cerebrovascular accident; EuroSCORE II = European System for Cardiac Operative Risk Evaluation II; LBBB = left bundle branch block; LCA = left coronary artery; LVEF = left ventricular ejection fraction; LVOT = left ventricular outflow tract; MAC = mitral annular calcification; MI = myocardial infraction; NYHA = New York Heart Association; PPM = permanent pacemaker; RBBB = right bundle branch block; RCA = right coronary artery.

**Table 2:** Procedural characteristics and in-hospital complications.

	All Patients *n* = 129	PPM *n* = 15	No PPM *n* = 114	*p* Value
**Procedural characteristics**				
THV type				0.78
Evolut R	34 (26%)	5 (33%)	29 (25%)	
Evolut PRO	45 (35%)	5 (33%)	40 (35%)	
Evolut PRO+	50 (39%)	5 (33%)	45 (40%)	
THV size				0.81
23 mm	2 (2%)	0 (0%)	2 (2%)	
26 mm	23 (18%)	2 (13%)	21 (18%)	
29 mm	64 (50%)	7 (47%)	57 (50%)	
34 mm	40 (31%)	6 (40%)	34 (30%)	
Oversizing by annulus perimeter	17.8 [13.6, 21.9]	18 [10.6, 20.4]	17.7 [13.9, 22]	0.39
Oversizing by annulus area	45.2 [35.1, 57]	45.9 [28.2, 55.8]	44.11 [36, 57.1]	0.35
Balloon pre-dilation *n* (%)	89 (69%)	12 (80%)	77 (68%)	0.39
Capture–redeployment attempts *n* (%)	55 (42.6%)	6 (40%)	49 (43%)	0.83
Capture–redeployment numbers	2.3 ± 1.5	2.3 ± 1.5	2.3 ± 1	0.96
Balloon post-dilation	51 (40%)	8 (53%)	43 (39%)	0.27
Cusp overlap	83 (64%)	76 (67%)	7 (47%)	0.16
Implantation depth at NCC (mm)	3.8 [2.8, 4.3]	4.4 [4.1, 5.7]	3.6 [2.6, 4.1]	<0.001
MS length minus implant depth, (mm)	−0.6 ± 2.5	−3.9 ± 1.5	−0.3 ± 2.4	<0.001
ID > MS	64 (50%)	13 (87%)	51 (45%)	0.002
**In-hospital complications**				
In-hospital death	3 (2%)	0 (0%)	3 (3%)	>0.999
Periprocedural MI	0 (0%)	0 (0%)	0 (0%)	-
In-hospital stroke	5 (4%)	1 (7%)	4 (4%)	0.48
Vascular complications				
Major	0 (0%)	0 (0%)	0 (0%)	-
Minor	20 (16%)	2 (13%)	18 (16%)	>0.999
PVL ≥ moderate (echo)	8 (6%)	1 (7%)	7 (6%)	>0.999

Abbreviations: ID = implantation depth; MI = myocardial infraction; MS = membranous septum; NCC = non-coronary cusp; PVL = para-valvular leakage; THV = transcatheter heart valve.

**Table 3:** Univariate and multivariate analysis to identify predictors of conduction disturbances requiring PPM at 30 days.

Predictors	Univariate Analysis		Adjusted Regression Analysis	
	Odds Ratio (95% CI)	*p*-Value	Odds Ratio (95% CI)	*p*-Value
**Preprocedural aspects**				
EuroSCORE II	0.62 (0.40–0.95)	0.028		
RBBB	10.21 (3.01–239.8)	<0.001	26.343 (3.924–176.837)	0.001
Membranous septum length	0.34 (0.19–0.58)	<0.001	0.276 (0.132–0.576)	0.001
MAC ≥ moderate	3.12 (1.03–9.42)	0.043		
**Procedural aspects**				
Implantation depth at NCC	1.62 (0.16–2.25)	0.004	1.576 (1.020–2.435)	0.04
MS length minus implant depth	0.56 (0.41–0.76)	<0.001		

Abbreviations: EuroSCORE II = European System for Cardiac Operative Risk Evaluation II; MAC = mitral annular calcification; MS = membranous septum; NCC = non coronary cusp; RBBB = right bundle branch block.

**Table 4:** Change in PR interval and QRS duration measured pre-TAVI, post-TAVI and pre-discharge.

	Time	Median [IQR]	*p*-Value
PR interval (msec)	Pre TAVI *	182 [164, 209]	-
	Post TAVI	208 [178, 240]	0.002
	At discharge	188 [171, 219]	0.064
QRS dutaion (msec)	Pre TAVI *	101 [89, 119]	-
	Post TAVI	127 [107, 150]	<0.001
	At discharge	125 [100, 149]	<0.001

* Reference category. Abbreviations: IQR = interquartile range; msec = millisecond; TAVI = transcatheter aortic valve implantation.

## Data Availability

All data are contained within the article.
